# A “Bottom-Up” Approach to Aetiological Research in Autism Spectrum Disorders

**DOI:** 10.3389/fnhum.2013.00606

**Published:** 2013-09-19

**Authors:** Lisa M. Unwin, Murray T. Maybery, John A. Wray, Andrew J. O. Whitehouse

**Affiliations:** ^1^School of Psychology, The University of Western Australia, Perth, WA, Australia; ^2^Telethon Institute for Child Health Research, Centre for Child Health Research, The University of Western Australia, Perth, WA, Australia; ^3^Child and Adolescent Health Service, State Child Development Centre, Princess Margaret Hospital for Children, Perth, WA, Australia

**Keywords:** autism spectrum disorders, heterogeneity, autism phenotype

## Abstract

Autism spectrum disorders (ASD) are currently diagnosed in the presence of impairments in social interaction and communication, and a restricted range of activities and interests. However, there is considerable variability in the behaviors of different individuals with an ASD diagnosis. The heterogeneity spans the entire range of IQ and language abilities, as well as other behavioral, communicative, and social functions. While any psychiatric condition is likely to incorporate a degree of heterogeneity, the variability in the nature and severity of behaviors observed in ASD is thought to exceed that of other disorders. The current paper aims to provide a model for future research into ASD subgroups. In doing so, we examined whether two proposed risk factors – low birth weight (LBW), and *in utero* exposure to selective serotonin reuptake inhibitors (SSRIs) – are associated with greater behavioral homogeneity. Using data from the Western Australian Autism Biological Registry, this study found that LBW and maternal SSRI use during pregnancy were associated with greater sleep disturbances and a greater number of gastrointestinal complaints in children with ASD, respectively. The findings from this “proof of principle” paper provide support for this “bottom-up” approach as a feasible method for creating homogenous groups.

## Introduction

Autism spectrum disorders (ASD) are currently diagnosed in the presence of impairments in social interaction and communication, and a restricted range of activities and interests. However, there is considerable variability in the behaviors of different individuals with an ASD diagnosis. Traditionally, researchers have conceptualized ASD as a unitary disorder with a large spectrum, and have sought to discover a single aetiological factor that leads to disorder. However, the behavioral heterogeneity has been mirrored at the genetic level, for instance, many susceptibility loci have been identified, yet each has been found to account for a small amount of variance only (1–2%) (Weiss et al., 2008). A proposition that has gathered momentum over the last decade involves moving away from the traditional conceptualization of ASD as a unitary disorder toward conceptualizing a syndrome of multiple and separate disorders; in essence, re-examining “autism” as “the autisms” (Geschwind and Levitt, 2007; Whitehouse and Stanley, 2013).

Research in this area has traditionally adopted a “top-down approach” by constraining behavioral phenotypes in the hope that this will facilitate the identification of biological subtypes. For example, Buxbaum et al. (2001) reported linkage evidence for a susceptibility gene for Autistic Disorder on chromosome 2. In an analysis of 95 affected-relative pair families with Autistic Disorder they found a maximum multipoint heterogeneity LOD score (HLOD) of 1.96 and a maximum multipoint NPL score of 2.39 on chromosome 2q (at 186cM, for D2s364). When families were grouped according to delayed onset (at age >36 months) of phrase speech, linkage to chromosome 2 increased (HLOD = 2.99, NPL = 3.32). Shao et al. (2002) found further evidence for a susceptibility gene on chromosome 2. In an analysis of 82 sibling pairs with Autistic Disorder they found a HLOD of 0.53 at D2S116. When the analysis was restricted to a subset of 45 families with phrase speech delay (>36 months), linkage to chromosome 2q increased (HLOD = 2.12). Whilst this approach has received the most attention in aetiological research, generally speaking, it has underperformed, with only weak evidence that stratification based on IQ, age at first word, or verbal ability yield a more genetically homogenous population (Geschwind and Levitt, 2007).

A “bottom-up” approach to identify biological subtypes of ASD has not received the same level of research attention. This methodology focuses on known aetiological risk factors, and whether individuals exposed to these risk factors have a more homogenous phenotype. In this paper, we report on this bottom-up approach, focusing on aspects of the phenotype that are not part of the core defining features of the disorder. We know that comorbid medical conditions are highly prevalent in ASD (Bauman, 2010). Sleep problems are thought to affect 40–80% of children on the spectrum (Richdale, 1999) and estimates of gastrointestinal disorders in ASD range from 9 to 70% (Buie et al., 2010). The high prevalence of these comorbid conditions in children with ASD may suggest the presence of important genetic and/or biological markers, which if identified, can refine our ability to be more precise in categorizing clinical and genetic subtypes within the autism spectrum (Bauman, 2010). In this paper, we have adopted a bottom-up approach by stratifying groups based on two previously identified risk factors, namely, maternal use of selective serotonin reuptake inhibitors (SSRIs) during pregnancy and low birth weight (LBW). The second part of our strategy involved examining the homogeneity within the groups based on medical complaints such as sleep problems and gastrointestinal complaints in addition to core features of ASD such as social behavior, language characteristics, and severity.

Selective serotonin reuptake inhibitors use during pregnancy has gained considerable attention over the last 2 years and is thought to be implicated in an increased risk of ASD diagnosis (Croen et al., 2011). Prevalence studies in the US estimate that up to 8% of mothers may be treated with SSRIs during pregnancy for conditions such as anxiety disorders or major depression (Alwan et al., 2011). SSRIs act primarily by blocking the serotonin transporter, thereby raising extracellular serotonin (5-HT) levels (Oberlander et al., 2009). These SSRIs readily cross the placental and blood-brain barriers to the fetus, with the potential to alter central 5-HT signaling (Oberlander et al., 2009). The neuroactive properties of SSRIs are thought to be a potential risk to fetal neurodevelopment, since 5-HT plays such a critical role in regulating diverse processes such as cell division, differentiation, migration, myelination, synaptogenesis, and dendritic pruning (Gaspar et al., 2003). A number of researchers have hypothesized that the increase in ASD diagnoses in recent years may be associated with a commensurate increase in maternal use of antidepressant medication during pregnancy (Croen et al., 2011). A recent population-based case-control study by Croen et al. (2011) reviewed record-based data describing the postnatal development of children exposed to SSRIs *in utero*. This study examined data for children born through a medical care program during the period of 1995–1999. Infants in the sample who were later diagnosed with ASD were considered cases. Children without an ASD diagnosis were randomly sampled from the remaining cohort at a ratio of five control children per one case child. Using this matched sample, Croen et al. (2011) investigated SSRI use throughout pregnancy and found that 70 women who took antidepressant medication the year before the birth of their child had twice the risk of having a child with ASD (*n* = 20 offspring with ASD, 28.57%) compared with 1735 women who did not take any antidepressant medication (*n* = 278 offspring with ASD, 16.02%).

Using a similar population-based nested case-control design, Rai et al. (2013) investigated the extent to which parental depression and maternal antidepressant use during pregnancy were associated with ASDs in offspring. For parental depression, record-based data was available for 4429 cases of ASD and 43277 age- and sex-matched controls, and for maternal antidepressant use, data existed for 1679 ASD cases and 16845 non-ASD controls. They found that a history of maternal but not paternal depression was associated with higher risk of autism in offspring. These associations were largely limited to children of mothers who reported using antidepressants at the first antenatal interview. Antidepressant use during pregnancy was reported by 1.3% of mothers of children with ASD and by 0.6% of control mothers, equating to an almost twofold increase in risk of ASD with use of antidepressants (Rai et al., 2013). Other studies that have examined the effect of maternal SSRI use during pregnancy have observed several atypical behavioral outcomes among offspring, including delay in meeting gross motor milestones (Pedersen et al., 2010), a wide range of feeding difficulties (Oberlander et al., 2006) and sleep disturbances (Zeskind and Stephens, 2004).

Low birth weight (<2500 g) has also been considered an environmental risk factor implicated in a range of psychiatric disorders including ASD, anxiety disorder, and depression (Indredavik et al., 2004; Gardener et al., 2011; Jaspers et al., 2012). Lampi et al. (2012) examined data from the case-control Finnish Prenatal Study of Autism and ASDs and found that children with very LBW (<1500 g) had a greater than threefold increased odds of autism compared with children with normal birth weight (NBW) (2500–3999 g). Interestingly, LBW did not significantly increase the odds of Asperger syndrome (Lampi et al., 2012). In addition to these associated psychiatric disorders, when compared to children with NBW, children with LBW have been found to show pervasive motor impairments, increased socio-emotional issues, increased risk of sleep-disordered breathing, and reductions in language ability (Paavonen et al., 2007; de Kieviet et al., 2009; Spittle et al., 2009; Barre et al., 2011; Scott et al., 2012). Despite evidence supporting the role of these environmental risk factors in the development of ASD, no single factor has been identified that poses a determinant risk for this disorder.

This paper will adopt a “bottom-up” approach to parsing ASD heterogeneity by investigating the behavioral phenotype associated with two possible environmental risk factors. The first study compared the behavioral and developmental phenotype of children with ASD whose mothers used SSRIs during pregnancy with the phenotype for a tightly matched group of children with ASD whose mothers did not use SSRIs during pregnancy. It was hypothesized that those children with ASD whose mothers used SSRIs during pregnancy would display early feeding and sleep disturbances compared to the control group of children with ASD. We also examined whether these children showed a distinguishable behavioral phenotype. Study 2 compared the phenotype of children with ASD born with LBW with a matched group of children with ASD born with NBW. It was hypothesized that those LBW children with ASD would display greater sleep disturbances (e.g., sleep-disordered breathing), language difficulties, and socio-emotional problems compared to the NBW group. This “proof of principle” study seeks to examine two potential risk factors within the context of a “bottom-up” research design. If the hypotheses are supported this paper may provide a blueprint for using the “bottom-up” approach as a feasible method for creating homogenous groups compared with the more costly “top-down” approach which requires large sample sizes.

## Study 1

### Materials and Methods

#### Participants

Participants were part of the Western Australian Autism Biological Registry (WAABR), which is an ongoing study of children with a clinical diagnosis of an ASD and their families taking place at the Telethon Institute for Child Health Research in Perth, Western Australia (see Taylor et al., in press). Diagnosing ASD in Western Australia mandates assessment by a clinical team comprising a Pediatrician, Psychologist, and Speech-Language Pathologist under DSM-IV guidelines (American Psychiatric Association, 1994). A diagnosis is only made when there is consensus amongst the team. The current study included nine participants from the WAABR whose mothers reported SSRI use during pregnancy (cases). Each of these participants was individually matched on gender and chronological age at assessment (within 15 months) with three further children with ASD (*n* = 27) whose mothers did not take an SSRI during pregnancy.

#### Measures and procedure

Prior to attending a face-to-face assessment, families were mailed and asked to complete a comprehensive case-history questionnaire relating to the mother’s pregnancy and the ASD child’s development. Mothers were asked to provide details of any history of psychological disorder such as major depression or anxiety. They were also asked to provide the name of any prescription or non-prescription medications, the dosage, and the amount they used during pregnancy. A series of questionnaires were also included in this package, including the Social Responsiveness Scale (SRS; Constantino and Gruber, 2002), Children’s Sleep Habits Questionnaire (CSHQ; Owens et al., 2000), Children’s Communication Checklist-2 (CCC-2; Bishop, 2003), and a gastrointestinal complaints questionnaire (Ibrahim et al., 2009).

The SRS is a 65-item questionnaire used to examine a range of social behaviors characteristic of ASD in children over the last 6 months. A total score can be calculated for the SRS as well as five subscale scores, namely, social communication, autism mannerisms, social motivation, social awareness, and social cognition. Parents respond using a four-point scale ranging from “not true” (1) to “almost always true” (4). A higher total score on this measure is indicative of greater social difficulties. The CSHQ is a 34-item parent-report instrument that was used to examine sleep behavior over a “typical week.” Parents were asked to rate how often their child showed behaviors such as “struggle at bedtime” and “show fear at sleeping alone” using a one to three point scale corresponding to “rarely,” “sometimes,” or “usually,” respectively. A total score and eight subscale scores (bedtime resistance, sleep onset delay, sleep duration, sleep anxiety, night wakings, parasomnias, sleep-disordered breathing, and daytime sleepiness) can be calculated for responses on the CSHQ. Higher total scores on the CSHQ indicate that the child has a greater number of sleep problems.

The CCC-2 is a parent-report questionnaire designed to assess the communication skills of children aged 4–16 years. The purposes of the CCC-2 are the identification of pragmatic language impairment, screening of receptive and expressive language skills, and assistance in screening for ASD. The CCC-2 consists of 70 items that are divided into 10 scales, each with 7 items. The first four scales focus on specific aspects of language and communications skills (content and form). The next four scales assess the pragmatic aspects of communication. The last two scales measure behaviors that are usually impaired in children with ASDs. The parent rates the frequency of the communication behavior described in each item from 0 (less than once a week or never) to 3 (several times a day or always). Interpretation is based on a General Communication Composite (GCC), with lower scores indicative of greater language and communication difficulties.

Parents also completed a brief questionnaire related to their child’s history of gastrointestinal problems. This questionnaire was developed specifically for the WAABR case-history questionnaire based on the list of complaints in Ibrahim et al. (2009). After reviewing the literature related to gastrointestinal symptoms they identified five categories that have been reported to be common in patients with autism, namely, constipation, diarrhea, gastro-esophageal reflux or vomiting, abdominal discomfort/irritability, or feeding issues (Ibrahim et al., 2009). If the parent reported their child had experienced any of the five gastrointestinal complaints for a period of at least a month, resulting in consultation with their doctor, they received a score of one for the indicated complaint(s). Any other reports received a score of zero. Using this scoring method these complaints were analyzed in two ways: (1) individually to see if the frequency of each complaint differed between the two groups and (2) as a summary measure of gastrointestinal complaints (score of one or more) versus no gastrointestinal complaints (score of zero).

Families were then invited to the Telethon Institute for Child Health Research for a face-to-face behavioral assessment. Clinical diagnoses of ASD were confirmed using the Autism Diagnostic Observation Schedule-Generic (ADOS-G; Lord et al., 2000). The present study used the child’s age and ADOS module (reflective of their quantity of speech) to calibrate severity scores (0–10) for each participant according to the severity scale of Gotham et al. (2009). This enabled comparisons between the participants, irrespective of the module they completed.

#### Statistical analyses

Between-group differences in the quantitative scores of the SRS, CCC-2, CSHQ, and ADOS severity scale were investigated with independent-samples *t*-tests. Responses to the gastrointestinal complaints questionnaire were analyzed according to whether parents reported zero complaints or one or more complaints for their children using chi-square analyses with Fisher’s exact test of significance.

### Results

The SSRI case (*n* = 9) and control (*n* = 27) groups did not significantly differ on gestational age [*F*(1, 34) = 1.05, *p* > 0.05] or maternal age at conception [*F*(1, 34) = 3.45, *p* > 0.05]. Table 1 provides details of the maternal, pregnancy and offspring characteristics of the case group.

**Table 1:** **Study 1: maternal and offspring characteristics of the SSRI case group**.

	Maternal			Offspring						
	SSRI taken during pregnancy	Period of pregnancy SSRI taken	Psychiatric diagnosis	Gestational age at birth (weeks)	Age at assessment	ADOS module administered	ADOS severity score	CSHQ score	SRS score	Number of gut problems
Case 1	Lexapro	Daily	Major depression	41	5, 6	2	1	42	146	2
Case 2	Lexapro	Daily	Major depression	40	4, 6	2	6	62	157	1
Case 3	Lovan	3 months	Major depression	36	5, 2	2	8	54	158	4
Case 4	Effexor	Daily	Major depression	38	10, 2	3	3	46	172	2
Case 5	Not specified	–	Major depression	38	4, 3	2	7	59	166	0
Case 6	Escitalopram	Daily	–	38	2, 9	1	4	77	207	1
Case 7	Fluoxetine	Daily	Anxiety disorder	40	8, 5	3	3	63	145	1
Case 8	Aropax	1 month	Anxiety disorder	39	3, 1	1	6	54	120	2
Case 9	Zoloft	Daily	Depression	38	3, 5	1	6	41	90	1

Independent-samples *t*-tests revealed that there were no significant differences between the two groups on any of the SRS, CCC-2, CSHQ, or ADOS severity scores (Table 2). However, analysis of responses to the gastrointestinal complaints questionnaire found that mothers who used SSRIs during pregnancy were more likely to have a child with ASD who experienced one or more gut problems (*n* = 8, 88.9%), compared to the control group (*n* = 13, 48.1%), χ^2^(1), *p* = 0.05. To further investigate this association, chi-square analyses with Fisher’s exact test were performed on the five individual complaints (Table 3). The individual complaints did not significantly differentiate between the groups. However, the percentage of constipation complaints was noticeably larger (though, not significantly) for cases compared to controls.

**Table 2:** **Study 1: descriptive statistics and independent-samples *t*-tests for CSHQ, SRS, and ADOS severity scores**.

	SSRI cases		Controls		Statistic	*p*
	*M*	SD	*M*	SD	
CSHQ	55.56	11.36	51.58	11.96	*t*(31) = 0.86	0.40
Bedtime resistance	9.56	2.79	9.83	3.25		
Sleep onset delay	1.89	0.60	1.83	0.87		
Sleep duration	5.11	2.42	4.96	2.39		
Sleep anxiety	4.89	1.45	4.79	1.82		
Night wakings	6.00	1.50	5.04	2.03		
Parasomnias	11.33	2.78	9.58	3.36		
Sleep dis-breathing	4.11	1.83	4.08	1.56		
Daytime sleepiness	12.67	3.16	11.46	2.95		
SRS	151.22	32.84	145.11	25.86	*t*(34) = 0.57	0.57
Social awareness	16.56	1.81	16.44	2.94		
Social cognition	28.78	8.23	26.56	6.24		
Social communication	50.33	12.58	47.74	8.88		
Social motivation	25.56	5.36	24.93	5.95		
Autistic mannerisms	30.00	7.78	29.44	7.54		
ADOS severity	4.89	2.26	5.93	1.96	*t*(34) = 1.32	0.19
CCC-2
GCC	30.75	6.99	36.15	16.53	*t*(15) = 0.63	0.54

**Table 3:** **Study 1: chi-square analyses using Fisher’s exact test for both groups of children for the five gastrointestinal complaints**.

Gut complaint	SSRI cases *N* (%)	Control *N* (%)	*p*
Constipation	4 (44.4)	4 (14.8)	0.09
Diarrhea	2 (22.2)	3 (11.1)	0.58
Gastro reflux	2 (22.2)	3 (11.1)	0.58
Abdominal	1 (11.1)	2 (7.4)	1.00
Feeding	5 (55.6)	8 (29.6)	0.24
One or more complaints	8 (88.9)	13 (48.1)	0.05

### Discussion

This is the first study to examine the relationship between SSRI exposure and ASD phenotype. There were no differences between the cases and individually matched control participants in scores on the SRS, CCC-2, CSHQ, or ADOS-G severity. However, children with ASD whose mothers took SSRIs during pregnancy were significantly more likely to experience gastrointestinal complaints during childhood. Further examination of the relationship between gastrointestinal complaints and *in utero* SSRI exposure revealed that no individual complaint could significantly differentiate the two groups. While this does not support Oberlander et al. (2009) who found evidence for feeding disturbances in typically developing infants exposed to SSRIs *in utero*, it is possible that the small sample size contributed to the null findings for the less-frequent individual complaints.

The current study was limited by the absence of a control group of children whose mothers had affective disorders but who did not take SSRIs during pregnancy, and therefore we are unable to parse out whether the differences in the frequency of gut problems is related to mood disturbances or SSRI use. Rai et al. (2013) reported an association between maternal depression and an increased risk of offspring ASD. Although they found that this association was largely confined to antidepressant use in a subsample of mothers, future studies could build on the findings presented here and in Rai et al. (2013) by comparing the phenotype for children with ASD whose mothers report untreated depression during pregnancy with a matched ASD control group of children. The hypothesized association between ASD and gastrointestinal pathology is the subject of increasing amounts of research. Despite the numerous parental reports of gastrointestinal complaints among their children with ASD, studies have failed to find a significant difference in the prevalence of these complaints between children with ASD and control groups of children (e.g., Ibrahim et al., 2009). The current findings suggest that SSRI exposure *in utero* may be one potential candidate accounting for variance in the gut phenotype in children diagnosed with ASD.

## Study 2

### Materials and Methods

#### Participants

The study involved using data for 16 participants from WAABR whose birth weight was ≤2500 g (LBW). Each of these participants was individually matched on gender and chronological age at assessment (within 18 months) with two further control children with ASD (*n* = 32) whose birth weight was within the normal range (NBW; 2500–3999 g).

#### Measures and procedure

Within the case-history questionnaire, mothers were asked to report their child’s birth weight. For the purposes of Study 2, data collected for each child using the SRS, CSHQ, ADOS severity, CCC-2, and gastrointestinal complaints questionnaire were analyzed.

#### Statistical analyses

Between-group differences in the quantitative scores of the SRS, the CSHQ, CCC-2, and ADOS severity scale were investigated with independent-samples *t*-tests. Responses to the gastrointestinal complaints questionnaire were analyzed using chi-square analyses with Fisher’s exact test of significance.

### Results

The LBW (*n* = 16) and the NBW (*n* = 32) groups did not significantly differ on maternal age at conception [*F*(1, 45) = 0.07, *p* > 0.05]. Mean gestational age was significantly lower for the LBW group [*F*(1, 43) = 28.53, *p* < 0.05, *M* = 34.25 weeks, SD = 4.55 weeks] relative to the NBW group (*M* = 39.07 weeks, SD = 1.33 weeks, *p* < 0.05). Table 4 provides details of the offspring characteristics of the case group. Independent-samples *t*-tests (see Table 5) revealed that LBW children with ASD had significantly higher scores relative to the NBW group on the CSHQ for Total Sleep Disturbance and two of the subscales, namely, Sleep-Disordered Breathing and Daytime Sleepiness. There were no significant differences between the two groups on the SRS, CCC-2, or ADOS severity scores (Table 5).

**Table 4:** **Study 2: offspring characteristics of the LBW case group**.

	Birth weight	Gestational age at birth	Age at assessment	ADOS module	ADOS severity score	CSHQ score	SRS score	GCC	Number of gut problems
Case 1	600	24	11; 1	3	4	45	152	42	1
Case 2	895	27	7; 4	2	6	57	166	32	3
Case 3	985	29	5; 6	1	3	39	133	38	1
Case 4	1565	30	4; 7	1	6	42	172	–	0
Case 5	1640	37	5; 2	1	6	65	169	40	2
Case 6	1665	37	5; 2	2	4	56	143	49	1
Case 7	1725	35	14; 4	3	4	63	171	28	3
Case 8	1765	34	2; 8	1	7	47	107	–	1
Case 9	2097	40	13; 1	1	6	54	183	–	2
Case 10	2285	36	5; 2	2	8	54	158	–	4
Case 11	2300	37	5; 11	1	7	51	163	–	0
Case 12	2426	37	9; 7	1	8	69	200	-	1
Case 13	2450	32	4; 7	2	6	52	159	56	1
Case 14	2500	38	4; 3	2	7	59	166	39	0
Case 15	2125	37	11; 3	3	6	66	191	7	1
Case 16	2480	38	4; 6	2	5	66	156	–	2

**Table 5:** **Study 2: descriptive statistics and independent-samples *t-*tests for CSHQ, SRS, CCC-2, and ADOS severity score**.

	LBW		NBW		Statistics	*p*
	*M*	SD	*M*	SD	
CSHQ	55.31	9.08	47.84	8.84	*t*(45) = 2.72	0.01
Bedtime resistance	9.81	2.76	8.13	2.38		
Sleep onset delay	1.88	0.72	1.52	0.68		
Sleep duration	5.69	2.39	4.55	1.93		
Sleep anxiety	4.63	1.63	4.74	1.86		
Night wakings	5.19	1.87	4.19	1.66		
Parasomnias	10.44	2.71	9.97	2.56		
Sleep dis-breathing	4.31	1.49	3.29	0.82	*t*(45) = 3.04	0.00
Daytime sleepiness	13.38	3.36	11.19	2.56	*t*(45) = 2.48	0.02
SRS					*t*(45) = 0.75	0.46
Social awareness	17.31	2.73	17.97	2.33		
Social cognition	30.25	3.64	28.77	5.81		
Social communication	54.00	8.25	51.13	8.58		
Social motivation	27.69	4.70	26.68	4.88		
Autistic mannerisms	32.56	7.16	31.68	7.36		
ADOS severity	5.81	1.47	6.56	1.98	*t*(46) = 1.34	0.19
CCC-2
GCC	36.78	13.92	28.88	13.67	*t*(31) = 1.47	0.15

Similarly, children with LBW (*n* = 16, 81%) did not experience significantly greater gastrointestinal issues compared to the NBW group (*n* = 32, 53%), χ^2^(1), *p* = 0.07. To further investigate this association, chi-square analyses with Fisher’s exact test were performed on the five individual complaints (Table 6). The individual complaints did not significantly differentiate between the groups.

**Table 6:** **Study 2: chi-square analyses using Fisher’s exact test for both groups of children for the five gastrointestinal complaints**.

Gut complaint	LBW *N* (%)	NBW *N* (%)	*p*
Constipation	7 (43.8)	9 (28.1)	0.22
Diarrhea	2 (12.5)	3 (9.4)	0.55
Gastro reflux	5 (31.3)	8 (25)	0.45
Abdominal	0 (0)	4 (12.5)	0.19
Feeding	9 (56.3)	13 (40.6)	0.24
One or more complaints	8 (88.9)	13 (48.1)	0.05

### Discussion

The second study examined the phenotype of children with ASD born with LBW relative to a group of children with ASD born with NBW. This study did not find any significant differences between the groups on the gastrointestinal complaints questionnaire, SRS, ADOS-G severity, or CCC-2. This is inconsistent with findings of greater socio-emotional issues and reduced language ability in LBW children compared to NBW children in the absence of an ASD diagnosis (Barre et al., 2011; Scott et al., 2012). The present study did find that children in the LBW group obtained higher mean scores on the CSHQ for total sleep disturbance, Daytime Sleepiness, and Sleep-Disordered Breathing relative to the NBW group. This supports the finding of sleep-disordered breathing in children with LBW without an ASD diagnosis (e.g., Paavonen et al., 2007). Interestingly, compared to norms from typically developing children (*M* = 3.24) and children with ASD (*M* = 3.92) (Hoffman et al., 2006), LBW children with ASD obtained larger mean scores for the Sleep-disordered Breathing subscale (*M* = 4.31).

Currently, there are no norms to describe performance of typically developing LBW children on the CSHQ. It would be interesting to compare sleep disturbance between LBW typically developing children and LBW children with ASD. Thus it may be useful to conduct a more comprehensive study of LBW and NBW children with and without ASD to look more closely at the significance of the present findings. Unsurprisingly, the LBW children had a significantly lower gestational age at birth than the NBW children, which raises the possibility that gestational age may be driving the findings and not birth weight. However, it is important to note that the study by Lampi et al. (2012), which informed our hypotheses, found that LBW was a better predictor of ASD diagnosis than was prematurity.

## General Discussion

This present study used a “bottom-up” approach to seek understanding of the heterogeneity of ASD by investigating the behavioral phenotype associated with two suspected environmental risk factors, namely, *in utero* SSRI exposure and LBW. It was hypothesized that children with ASD who were exposed to one of these environmental risk factors would present with a more homogenous phenotype relative to individually matched control groups of children with ASD. There was some preliminary support for this hypothesis. While the children in the LBW and SSRI-exposed groups were no different to their respective control groups in quantitative and qualitative measures of the core symptomatology of autism, there was evidence that the two groups were distinct in the level of their non-core symptomatology such as sleep and gastrointestinal complaints, respectively.

The numbers of children with ASD in the “aetiological risk” subgroups are small, and therefore we urge caution in drawing conclusions from these data. Rather, we seek to highlight a different method for understanding the heterogeneity in the ASD phenotype. We believe that the preliminary findings of increased levels of non-core symptoms of ASD among certain “aetiological risk” subgroups, provides evidence that this “bottom-up” methodology may assist ASD research. Studies including larger samples of children with ASD will build on the research presented here, and provide the opportunity to validate our preliminary findings.

Whilst the present study did not find any differences in core ASD symptoms between LBW and SSRI-exposed children with their respective control groups, we know that each child who is given an ASD diagnosis presents with the triad of core symptoms irrespective of their severity. It is unlikely that a single environmental factor could be attributed to “causing” one of these core impairments. Rather we may expect that the interplay between the environment and a child’s genetic profile contributes to the variable expression of autistic-related traits (Ratajczak, 2011). Therefore, it seems reasonable that environmental factors may be related to the expression of non-core ASD symptoms among these children rather than to any variance in core symptomatology.

Recently, Whitehouse and Stanley (2013) reaffirmed an emerging view in the literature with regard to reconceptualizing autism in moving away from a unitary disorder with one cause, and toward an “umbrella” for a collection of behavioral disorders resulting from a range of causal pathways. In their paper they describe how research in cerebral palsy may be analogous to research on autism. Initially cerebral palsy was thought to be a unitary disorder caused by anoxia secondary to trauma occurring during labor and delivery. However, the heterogeneity in symptoms and severity amongst children with cerebral palsy led researchers to hypothesize that there may be many causal pathways. Many other causes were identified for cerebral palsy following this reconceptualization, such as complications of preterm birth, infections, and inflammation *in utero* (McIntyre et al., 2012). For diagnosis, cerebral palsy is now considered an umbrella term covering a wide range of syndromes that arise secondary to a number of brain lesions/anomalies occurring early in development (Badawi et al., 2008).

A key question facing the field is whether the long-held view that autism is a unitary disorder with a single causal pathway is correct, or whether autism may best be conceptualized as an umbrella term for a collection of behavioral disorders resulting from a range of causal pathways, analogous to cerebral palsy. Current evidence suggests that the latter may be a more accurate representation. Heterogeneity in the distal causes of autism is now well-established. It is estimated that between 10 and 15% of individuals with autism have a known genetic aetiology, but the loci and nature of these lesions vary, from known syndromes to observable cytogenetic lesions and rare *de novo* mutations (e.g., copy number variations) (Abrahams and Geschwind, 2008). Among those with idiopathic autism, no single genetic risk variant has been found to occur in more than 1% of individuals (Abrahams and Geschwind, 2008). Similarly, environmental risk factors identified through epidemiological studies and examined in this study – *in utero* exposure to SSRIs (Croen et al., 2011) and LBW (Lampi et al., 2012) – differ considerably in the hypothesized biological paths to disorder, and as yet, no known environmental exposure is deterministic of autism.

Given that diagnosis is currently based on behavior, the question of whether autism is one or multiple disorders is ultimately a query over the proximal causes of these behaviors, and one perhaps best addressed in neuroscience. Neuroscientific studies may help determine whether (a) distal risk factors “fan in” on a common neurobiological substrate that has the capability of underpinning the considerable behavioral heterogeneity in autism (one disorder), or (b) the exact combination of distal risk factors determines the brain regions and functions that are affected, which in turn prescribe the behavioral profile of each individual (multiple disorders). A key research aim will be to investigate the correspondence (if any) between known distal (genetic and environmental) and proximal (neurobiological) risk factors for autistic behaviors, using increasingly sophisticated environmental monitoring, genetic sequencing, and neuroimaging techniques.

Using preliminary data in this study we have demonstrated how a “bottom-up” approach can be applied to current aetiological research. Grouping individuals using this method may facilitate the identification of subtypes of people with ASD. Elucidating the underlying nature of the disorder(s) is a crucial step toward achieving perhaps the “holy grail” of autism research: tailoring intervention to the biological and cognitive makeup of each individual (Whitehouse and Stanley, 2013).

## Conflict of Interest Statement

The authors declare that the research was conducted in the absence of any commercial or financial relationships that could be construed as a potential conflict of interest.
